# Multiple transitions between normal and hyperballistic diffusion in quantum walks with time-dependent jumps

**DOI:** 10.1038/s41598-019-55642-5

**Published:** 2019-12-17

**Authors:** Marcelo A. Pires, Giuseppe Di Molfetta, Sílvio M. Duarte Queirós

**Affiliations:** 1 Centro Brasileiro de Pesquisas Físicas, Rua Dr Xavier Sigaud, 150, 22290-180 Rio de Janeiro, RJ Brazil; 2 Aix-Marseille Université, Université de Toulon, CNRS, LIS, Natural Computation research group, Marseille, France; 3 Associate to the National Institute of Science and Technology for Complex Systems, Rio de Janeiro, Brazil

**Keywords:** Theoretical physics, Complex networks

## Abstract

We extend to the gamut of functional forms of the probability distribution of the time-dependent step-length a previous model dubbed *Elephant Quantum Walk*, which considers a uniform distribution and yields hyperballistic dynamics where the variance grows cubicly with time, *σ*^2^ ∝ *t*^3^, and a Gaussian for the position of the walker. We investigate this proposal both locally and globally with the results showing that the time-dependent interplay between interference, memory and long-range hopping leads to multiple transitions between dynamical regimes, namely ballistic → diffusive → superdiffusive → ballistic → hyperballistic for non-hermitian coin whereas the first diffusive regime is quelled for implementations using the Hadamard coin. In addition, we observe a robust asymptotic approach to maximal coin-space entanglement.

## Introduction

In his seminal article on quantum computing, Richard Feynman^1^ suggested computers which use quantum logic for information processing may be employed to simulate quantum systems efficiently, even when that is impossible to computers based on classical logic. To simulate the dynamics of a quantum system usually means to describe the system in terms of qubits — as well as its dynamics — by a succession of local and unitary operations, involving at most two qubits at time. In recent decades, Quantum Walks (QWs) — or its multi-particle generalization —, namely quantum cellular automata, have become the most natural way to design a wide range of complex phenomena and extensively used for their comprehension. QWs are frequently translated into simple models which act as proxies for rather complex dynamics as those governed by quantum fields. Besides offering easily implementable physical protocols, such approach has opened new avenues for the fundamental understanding of those processes. For this reason, research in quantum walks has bridged disciplines such as natural calculus and algorithmics^2–5^, quantum field theory^6–9^ and discrete geometry^10–12^, complex systems^13–15^ and machine learning^16,17^. Formally, a QW describes the unitary dynamics of one quantum particle and its internal degrees of freedom. The key content of a discretisation unit — or’cell’ — is whether or not the particle occupies that cell and what its internal state is. Moreover, as for any quantum system, these properties may be found in superposition. In a single time step the particle can only move a finite distance^18–20^. That protocol was then systematically extended on graphs^21^ and later fully mathematically examined by^22^.

A significant interpretation of QWs concerns looking at them as mathematical frameworks to study dissipative quantum computing/simulation^23,24^. Explicitly, in most of the cases which are worth studying — namely those related to small/nano-systems —, the noise level is high. Thus, if purely coherent quantum computing is a long standing goal, the modelling and simulation of efficiently noisy quantum systems is a mid-term objective and thus a relevant milestone in that research path. Few, yet important, results have been obtained in this direction, mainly focussing on one particle models, which although rich, may still have inherent limits; for instance, the authors in ref. ^25^ have generalised the usual noisy unitary QW to the so-called Open QWs, in order to describe the environment interaction and^26^ has recently understood to what extent the environmental noise can enhance quantum transport in photosynthetic complexes, i.e., how mimicking natural open quantum systems (mostly biological systems) can optimise quantum information processing.

Complementary, it has surged an interest for super and hyper ballistic phenomena in some kind of physical systems, such as viscous electron flows and in some kind of disordered and quasiperiodic system^27^.

Within this same context the some of the authors (GDM and SMDQ) introduced^28^ an analytically treatable non-Markovian discrete time quantum walk in a one-dimensional lattice which yields hyper-ballistic diffusion that is characterised by a variance growing cubicly with time, *σ*^2^ ∝ *t*^3^. The key ingredient in that model is a temporal noise uniformly distributed in the translation operator. For its rules, that model can be understood as the quantum version of the classical non-Markovian’elephant random walk’ process^29^ and it was named ‘Elephant Quantum Walk’ after it.

In the following, we extend this class of Elephant Quantum Walks (EQW) to a more general family of the functional noise, namely the discretised *q*-exponential, so that a quite broad range of asymptotic behaviour can be analysed. For brevity, we call it generalised EQW or simply gEQW. That functional form bounded by both the delta-Dirac distribution — associated with the standard quantum walk — and the uniform distribution, which yields the elephant quantum walk.

## Results

We start by presenting our results for the diffusion of the packet that is computed from the second statistical moment at time *t*,

1 $${\overline{{x}^{2}}}_{t}=\sum _{x}\,{x}^{2}{P}_{t}(x)$$

where

2 $${P}_{t}(x)=|{\psi }_{t}^{L}(x){|}^{2}+|{\psi }_{t}^{R}(x){|}^{2}.$$

As we deal with initial conditions that yield a symmetric *P*_*t*_(*x*), it follows that $\overline{{x}^{2}}$ is indeed the variance ${\sigma }^{2}=\overline{{x}^{2}}-{\bar{x}}^{2}$ since $\bar{x}=0$.

**Figure 1:**
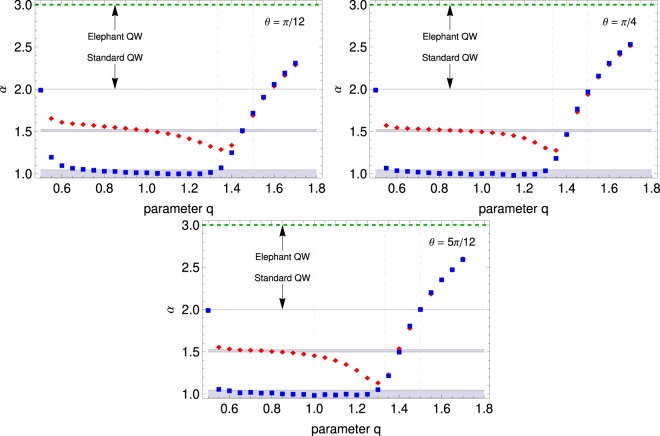
Dynamic regimes for $\theta =\{{15}^{o},{45}^{o},{75}^{o}\}$ the gEQW with ${\hat{C}}_{H}$ (red) and ${\hat{C}}_{K}$ (blue) coins. We use ${t}_{max}={2.10}^{4}$ to estimate *α* from $\overline{{x}^{2}} \sim {t}^{\alpha }$. Interestingly, jumps play a dual role in the QW: inhibition of wavepacket spreading for short-range steps and enhancement of spreading for long-range hopping.

To evaluate the dynamical regimes, we have computed the scaling exponent *α* of the power-law $\overline{{x}^{2}}\propto {t}^{\alpha }$, with $t\gg 1$. In Fig. 1, we show the gEQW exhibits a rich dynamics ranging from diffusive *α* = 1 to hyperballistic behaviour *α* = 3 for uniform (long-range) memory, which is approached as we increase the value of *q*, especially for $q\,\gtrapprox \,4/3$. On the other hand, it is visible the quantum standard walk can be understood a particular case in the relation between the functional form of the memory and the sort of diffusion performed by the walker because it corresponds to a singularity. In respect of that, we have performed a series of tests in the vicinity of *q* that confirmed our stance. We see jump-dependence functional form acts a double-edged sword: for $1/2 < q\,\lessapprox \,4/3$, the diffusion exponent decreases for *C*_*H*_ and remains largely diffusive using *C*_*K*_, whereas for $q\,\gtrapprox \,4/3$ one has an augment of *α*; moreover, the difference between the two coins vanishes that value of *q* on.

What is the reason for this twofold non-increasing/increasing behaviour of the diffusion exponent with *q*? At the fundamental level, we assert that it stems from the quantum superposition of the states during the evolution of the gEQW. We base our reasoning by computing the Relative Quadratic Deviation

3 $$RQD(x)\equiv {(x-\bar{x})}^{2}{P}_{t}(x),$$

where $\bar{x}=\sum _{x}\,x{P}_{t}(x)$, that in our case is null, $\bar{x}=0$, due to the symmetric nature of our *P*_*t*_(*x*). We regard *RQD*(*x*) as a local measure gauging the contribution of each site to the global variance.

**Figure 2:**
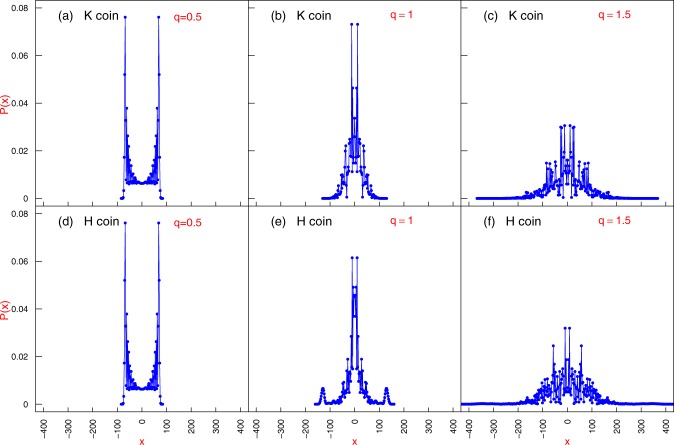
Probability distribution *P*_*t*_(*x*) at *t* = 100 for $\theta =\pi /4$. Panels show typical profiles for coins ${\hat{C}}_{H}$ and ${\hat{C}}_{K}$.

**Figure 3:**
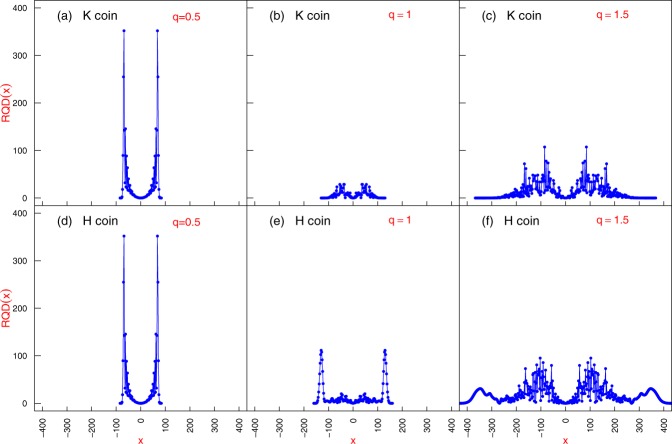
Local relative quadratic deviation *RQD*(*x*) at *t* = 100. Panels show typical profiles for the corresponding *P*_*t*_(*x*) in Fig. 2.

The results of *RQD*(*x*) and *P*_*t*_(*x*) for different values of *q* are plotted in Figs. 2 and 3 and interpreted as follows:

- for *q* = 1/2 — the memoryless case —, all the steps have the same size equal to 1. The Quantum Walker does not spread as much as the cases for *q* > 1/2, which leads to relatively small values of ${(x-\bar{x})}^{2}$. However, the distribution *P*_*t*_(*x*) exhibits peaks concentrated near the borders. This combination generates a *RQD*(*x*) profile with some large values, which are the key contributors to the ballistic spreading.
- For *q* = 1 — the exponential case—, we note that ${(x-\bar{x})}^{2}$ achieves higher values than in the previous case, because it is possible to get large steps that increase the occupation of positions *x* far away from the origin. Inasmuch as ${(x-\bar{x})}^{2}$ achieves larger values, *P*_*t*_(*x*) handicaps the sites near the borders giving rise to relatively small values of *RQD*(*x*). Conversely, the peaks of the probability near the origin are weakened by ${(x-\bar{x})}^{2}$ leading to small values *RQD*(*x*) as well;
- For *q* = 1.5 — in the asymptotic power-law regime — the wavepacket occupies substantially more sites; however, the site occupation is much less localised than in the preceding cases. The sites far off from the origin naturally have values of ${(x-\bar{x})}^{2}$ that are large enough to overcome its little probability *P*_*t*_(*x*). This sets forth a profile for *RQD*(*x*) that contains very large values, which induce the boosting of the gEQW.

**Figure 4:**
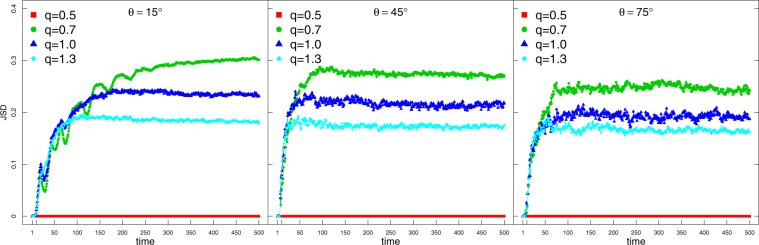
Time series for the Jensen-Shannon Dissimilarity (JSD) between ${P}_{t}^{H}(x)$ and ${P}_{t}^{K}(x)$ for typical angles $\theta =\{{15}^{o},{45}^{o},{75}^{o}\}$ and $q=\{0.5,0.7,1,1.3\}$.

In Fig. 3 we see the jumps give rise to a dissimilarity between the probability distributions obtained by utilising either the *H* or the *K* coin. In order to assess how this effect evolves we have computed the Jensen-Shannon dissimilarity (JSD)

4 $$JSD=\frac{KLD({P}^{h}|M)+KLD({P}^{k}|M)}{2}$$

where $M(x)=({P}^{h}(x)+{P}^{k}(x))/2$ corresponds to the point-wise midpoint distribution between the probability distributions *P*^*h*^(*x*) and *P*^*k*^(*x*). The JSD is based on a simmetrisation of the Kullback-Leibler dissimilarity (KLD) between two distributions *U* and *W*

5 $$KLD(U|W)=\sum _{x}\,{U}_{x}{\log }_{2}{U}_{x}/{W}_{x},$$

with the advantage that in the former the domain of distributions can be different without yielding an incommensurable result. In Fig. 4, we see that, as time elapses, the memoryless case (*q* = 0.5) is associated with similar distributions ${P}_{t}^{Hcoin}(x)$ and ${P}_{t}^{Kcoin}(x)$ and thus $JSD=0\,\forall t$. However, the introduction of memory changes that picture with noticeable sensibility of the probability distribution — hence the diffusion — to the coin operator.

In addition, we have evaluated the entanglement between the internal and external degrees of freedom (the so-called coin-space entanglement^30,31^) by means of the Von Neumann entropy

6 $${S}_{e}=-\,{\rm{Tr}}[{\rho }^{c}\,\log \,{\rho }^{c}],$$

where ${\rho }^{c}={{\rm{Tr}}}_{x}(\rho )$ is the reduced density matrix of the particle and *ρ* is the full density matrix $\rho =|\Psi \rangle \langle \Psi |$ of the QW system. An explicit expression was obtained previously (for instance see^31,32^),

7 $${\rho }^{c}(t)=[\begin{array}{ll}{G}_{a} & {G}_{ab}\\ {G}_{ab}^{\ast } & {G}_{b}\end{array}],$$

where ${G}_{a}=\sum _{x}\,|{\psi }_{t}^{L}(x){|}^{2}$, ${G}_{b}=\sum _{x}\,|{\psi }_{t}^{R}(x){|}^{2}$ and ${G}_{ab}=\sum _{x}\,{\psi }_{t}^{L}(x){({\psi }_{t}^{R}(x))}^{\ast }$. The entropy *S*_*e*_ is actually determined resorting to the eigenvalues *λ*^±^ of *ρ*^*c*^

8 $${S}_{e}=-\,{\lambda }^{-}\,{\log }_{2}{\lambda }^{-}-{\lambda }^{+}\,{\log }_{2}{\lambda }^{+}$$

9 $${\lambda }^{\pm }=\frac{1}{2}\pm \frac{1}{2}\sqrt{1-4{G}_{a}{G}_{b}+4|{G}_{ab}{|}^{2}}$$

**Figure 5:**
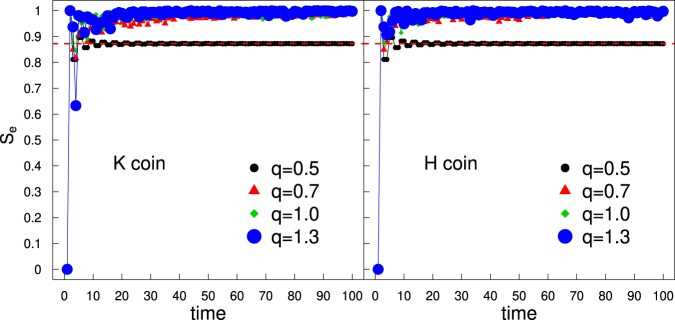
Time series for the Von Neumann entanglement entropy *S*_*e*_ for $\theta =\pi /4$. The horizontal dashed red line corresponds to ${S}_{e}=0.872\,\ldots$ for the standard QW first numerically obtained in^30^ and later analytically demonstrated in^31^.

Interestingly, in Fig. 5 we can understand that for all *q* > 1/2 one finds a very large entanglement entropy *S*_*e*_ ≈ 1 in the long-rung. Similar results were obtained in ref. ^32,33^ however, the authors assumed a time-dependent disorder in the coin operator. But epistemologically, both model fits within the class of quantum walks with the temporal disorder.

## Discussion

We have comprehensively explored an extension of the EQW, a non-Markovian quantum walk process where the jump sites are selected from a uniform distribution giving rise to hyperballistic diffusion. Even though jumps in QWs have been introduced either in discrete-time^34–40^ and continuous-time^41–45^ models, we highlight that none of these previous works have found the richness of dynamical transitions we have presented herein with our new time-dependent protocol of jumps and two types of coin operators. Our flexible distribution, the *q*-exponential, allows us to recover both the standard QW in the limit *q* → 1/2 and the previous proposal *q* → *∞*^28^, providing a more general framework.

Since the model is about a walker, we first focussed on its diffusion properties. We have verified that the functional form of the distribution from which the jumps are drawn impacts in the kind of diffusion exhibited by the model. Particularly, for ${q}^{\ast }\lesssim 4/3$ the walker is sub-ballistic with specific behaviour depending on the sort of quantum coin that is applied. Concretely, setting apart the case *q* = 1/2 for which the standard QW is retrieved whatever the case, for the Hadamard-type coin the diffusion slowly decays from an exponent value slightly larger than the superdiffusion case *α* = 1.5 and from q* onwards the dynamics changes to a consistent increase of the diffusion exponent with *q* until it reaches the hyperballistic regime when *q* → *∞*. On the other hand, the non-hermitian coin yields a normal diffusive regime — within statistical significance — for *q* < q* and therefrom the value diffusion exponent soars up to the EQW case. In other words, in the scenarios of small range hopping the H coin provides a new mechanism for anomalous diffusion^46^. We assign to the eigenvalue structure introduced by *C*_*H*_ and *C*_*K*_ the difference in the diffusion behaviour that is developed in each case.

Although we did not manage to find an analytical evidence thereof, we cannot help mentioning the values *q* whereat the model both starts to augment *α* and recovers ballistic diffusion (with *q* ≠ 1/2) evenly match the cases in which *q*-exponential distribution stops having finite standard deviation and average, respectively. Nevertheless, resorting to a simple measure derived from the variance we have been able to learn the dependence between the parameter *q* that defines the (asymptotic) functional form of the distribution and the diffusion exponent.

Concerning the entanglement produced in the model, the analysis of the density matrix eigenvalues we have verified a very large entanglement for all of the values of *q* = 1/2. Similar results were obtained in systems assuming disorder in the coin operator. Thus, we reckon that strong entanglement is a feature of QWs with some sort of randomness. At first, it seems counterintuitive that by introducing disorder it is possible to establish quite entangled states; however, it is actually this randomness that creates the change of overlap between the states so that the density matrix does not show a pure state structure. If we understand the emergence of entanglement as a manifestation of some kind of order — that can be in the form of propagation of information —, our results help characterise such class of models the quintessential complex system. In other words, we have microscopic details yielding a robust (and unexpected) macroscopic feature.

Taking into account Figs. 1 and 5, it is clear that our novel protocol allows controllability of the scaling behavior of the spreading while keeping a robust maximum asymptotic entanglement. This controlability is in strike difference with all the previous works with disorder either in the coin operator^32,33,47–55^ or in the step operator^37,38,40^ where there entanglement enhancement was only accompanied by an upper bounded ballistic spreading. Thus, we highlight that our protocol is the first that display both amplification of entanglement and tunable spreading from slower-than-ballistic to faster-than ballistic.

## Methods

We consider a QW over the (1 + 1)–spacetime grid. Its coin or spin degree of freedom lies in ${ {\mathcal H} }_{2}$, for which we may chose some orthonormal basis $\{|{v}^{L}\rangle ,|{v}^{R}\rangle \}$. The overall state of the walker lies in the composite Hilbert space ${ {\mathcal H} }_{2}\otimes { {\mathcal H} }_{{\mathbb{Z}}}$ and thus can be written as ${\Psi }_{t}=\sum _{x}\,{\psi }_{t}^{L}(x)|{v}_{L}\rangle \otimes |x\rangle +{\psi }_{t}^{R}(x)|{v}_{R}\rangle \otimes |x\rangle$, where the scalar field ${\psi }_{t}^{L}(x)$ (resp. ${\psi }_{t}^{R}(x)$) gives, at every position $x\in {\mathbb{Z}}$, the amplitude of the particle being there and about to move left (resp. right). We use $(t,x)\in {\mathbb{N}}\times {\mathbb{Z}}$, to label respectively instants and points in space and let:

10 $${\Psi }_{t+1}={W}_{t\text{'}}{\Psi }_{t}$$

where

11 $${W}_{t\text{'}}=\hat{S}(\hat{C}\otimes {{\rm{Id}}}_{{\mathbb{Z}}})$$

with $\hat{S}$ a state-dependent shift operator such that

12 $${({\hat{S}}_{{t}^{\text{'}}}\Psi )}_{t}(x)=(\begin{array}{l}{\psi }_{t}^{L}(x-{t}^{\text{'}})\\ {\psi }_{t}^{R}(x+{t}^{\text{'}})\end{array}).$$

where *t*′ represents the walker displacement on the lattice.

In the following we will consider two family of coin operators in *U*(2):

13 $$\begin{array}{cc}{\hat{C}}_{H}=(\begin{array}{ll}\cos \,\theta  & \sin \,\theta \\ \sin \,\theta  & -\,\cos \,\theta \end{array}) & {\hat{C}}_{K}=(\begin{array}{ll}\cos \,\theta  & i\,\sin \,\theta \\ i\,\sin \,\theta  & \cos \,\theta \end{array}).\end{array}$$

Respectively, the Hadamard-like coin ${\hat{C}}_{H}$ and a non-hermitian coins family ${\hat{C}}_{K}$ already introduced e.g. in^56^. We choose the following localised initial state:

14 $${\psi }_{0}^{L}(x)=\frac{1}{\sqrt{2}}{\delta }_{x,0},\,{\psi }_{0}^{R}(x)=\frac{{e}^{i\phi }}{\sqrt{2}}{\delta }_{x,0},$$

and, in order to have symmetric distributions, we will set $\phi =\pi /2$ for ${\hat{C}}_{H}$ and $\phi =0$ for ${\hat{C}}_{K}$.

In the family of EQWs introduced in^28^ the parameter *t*′ is the memory parameter, which at every interval [1, *t*] follows the functional form

15 $${{\mathscr P}}_{[1,t]}(k)\equiv {{\mathscr C}}_{t}{\exp }_{q}(\,-\,k)={{\mathscr C}}_{t}{[1-(1-q)\,k]}_{+}^{1/(1-q)},\,{\rm{with}}\,k=\{1,2,\,\ldots ,\,t\},$$

known as a *q*-exponential distribution as well. The quantity ${{\mathscr C}}_{t}$ is a time-dependent normalisation that must be updated at each iteration. Furthermore, from Eq. 15 we observe that as ${\rm{\min }}(k)=1\to \,{\rm{\min }}(t^{\prime} )=1$ then the minimum allowed step has length 1. Interpreting it as a kernel distribution, Eq. (15) has been applied in econometric^57^ as well as in cellular automata models^58^.

Looking at the functional form Eq. (15) we can identify the following traits: for *q* < 1, it has a’compact’ support (in the sense that for $t\gg 1$ the maximum value given by Eq. (15) is less than *t*), where the maximum value one can select is equal to the integer of $(\frac{1}{1-q})$; for *q* = 1, it gets an exponential form and is natural scale-dependence. For those two instances, all the statistical moments are finite (when *t* → *∞*). For *q* > 1, Eq. (15) exhibits an asymptotic power-law decay for which the *n*-th order statistical moment is not finite for $q\ge \frac{2+n}{1+n}$. That said, we understand that for *q* = 1/2 we will have the same dynamics as the standard Quantum Walk as we can only consider the nearest neighbour whereas the limit *q* → +*∞* concurs with the Elephant Quantum Walk case.
